# Supplementary material for: Implantable niche with local immunosuppression for islet allotransplantation achieves type 1 diabetes reversal in rats
Source: Nat Commun. 2022 Dec 26;13:7951. doi: 10.1038/s41467-022-35629-z (PMC9792517; doi:10.1038/s41467-022-35629-z)
Supplement: Supplementary file 1 — Supplementary Information [file 41467_2022_35629_MOESM1_ESM.pdf]

Supplementary Information

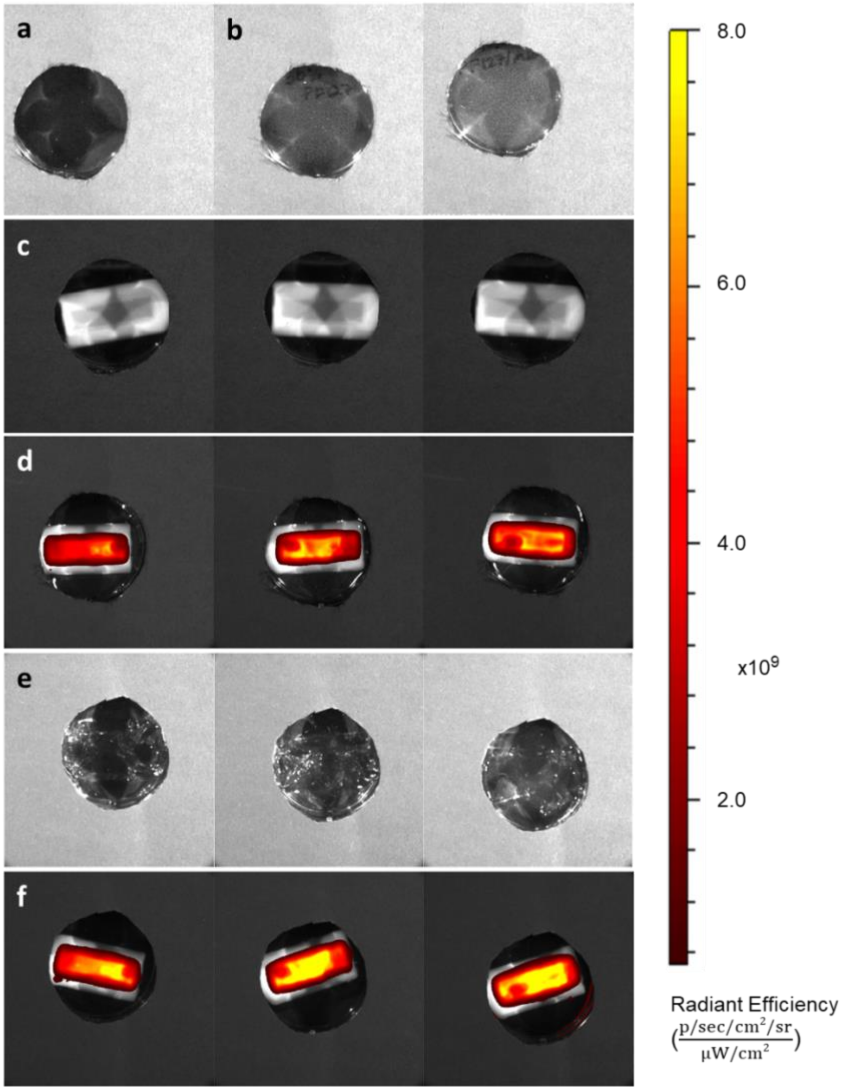

Supplementary Fig. 1 Assessment of cell permanence in NICHE. a Empty well, **b** 20% PF127, **c** Empty NICHE, **d** NICHE loaded with DiD-labeled MSCs after 24 h incubation, **e** Removed NICHE from original well, **f** NICHE in new well.

a

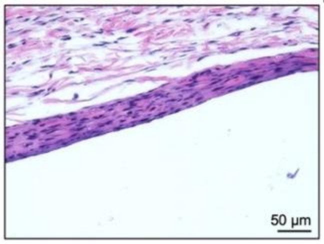

b

	Rat				NHP	
**Cell characteristic**	**Ti**	**Resin**	**PA 6wk**	**PA 12wk**	**Ti**	**PA**
Polymorphonuclear	0.0 ± 0.0	0.0 ± 0.0	0.0 ± 0.0	0.0 ± 0.0	1.7 ± 0.9	0.0 ± 0.0
Lymphocytes	2.4 ± 0.9	3.2 ± 1.1	2.5 ± 1.0	3.0 ± 1.0	2.5 ± 0.6	2.0 ± 0.0
Plasma cells	0.0 ± 0.0	0.0 ± 0.0	0.0 ± 0.0	1.3 ± 1.5	0.8 ± 0.3	0.0 ± 0.0
Macrophages	2.0 ± 1.4	2.0 ± 0.0	3.5 ± 1.0	3.3 ± 1.5	3.5± 0.6	2.0 ± 0.0
Giant cells	0.0 ± 0.0	1.6 ± 1.7	1.5 ± 1.0	1.3 ± 1.2	1.0 ± 0.9	1.0 ± 1.2
**Tissue characteristic**						
Necrosis	0.0 ± 0.0	0.0 ± 0.0	0.0 ± 0.0	0.0 ± 0.0	0.2 ± 0.3	0.0 ± 0.0
Capsule thickness	1.6 ± 0.5	2.0 ± 0.0	2.0 ± 0.8	1.7 ± 0.3	2.8 ± 0.4	1.3 ± 0.5
Tissue infiltrate	0.6 ± 0.5	0.4 ± 0.5	0.8 ± 0.5	0.0 ± 0.0	0.5 ± 0.4	0.0 ± 0.0
**Total**	6.6 ± 3.0	9.2 ± 1.6	10.3 ± 2.6	10.7 ± 4.4	13.0 ± 2.9	6.3 ± 1.5

c

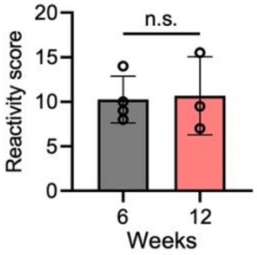

Supplementary Fig. 2 Tissue reactivity scoring. a Representative H&E staining from 5 biological replicates showing the fibrotic capsule formed around a titanium (Ti) device implanted in rats for 6-12 weeks. **b** Tissue reactivity scoring to Ti, resin, and nylon (PA) devices implanted in rats and nonhuman primates. c Tissue reactivity scoring to PA devices implanted in rats for 6 and 12 weeks (*n* = 4 independent animals), mean ± SD, unpaired two-tailed student's t-test n.s. *p* = >0.05.

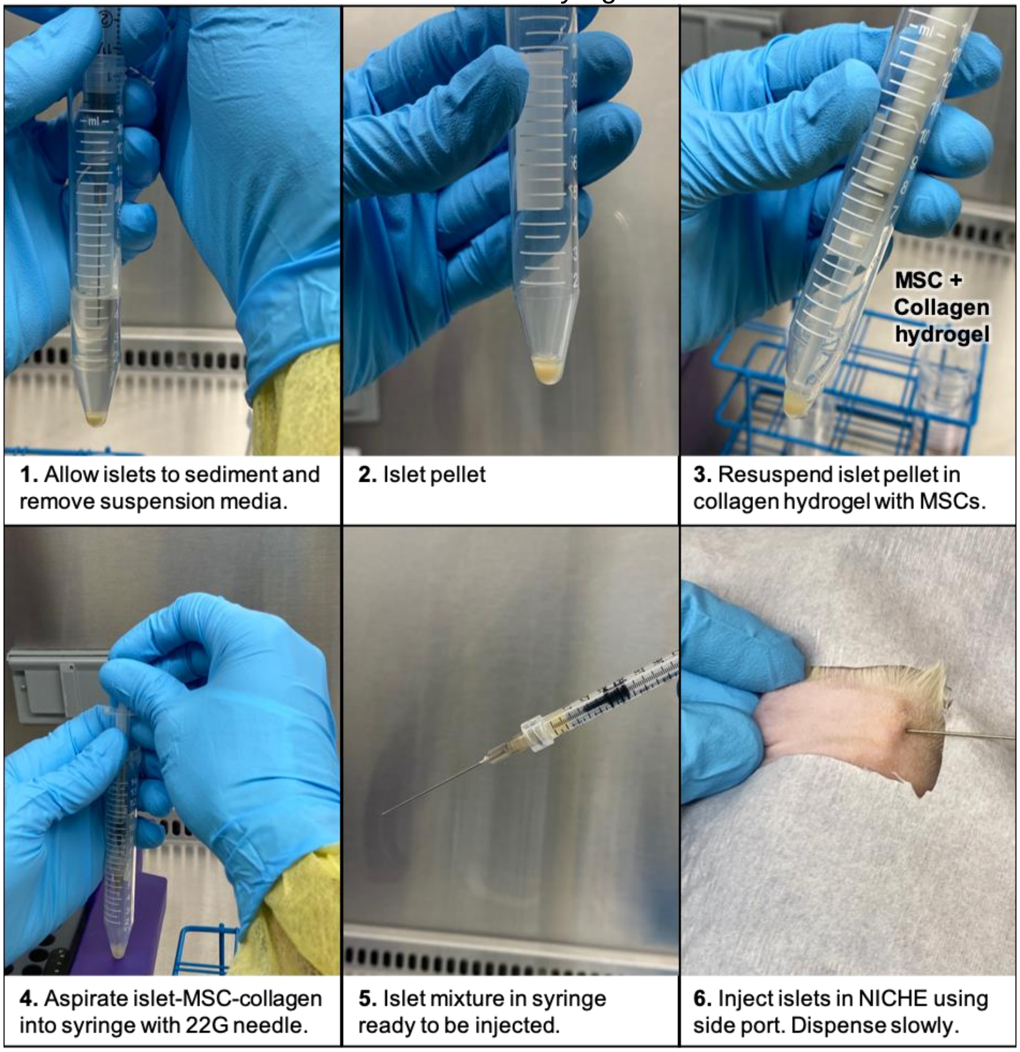

Supplementary Fig 3 Islet-MSCs transplantation procedure into the NICHE. Photographs showing the procedure for islet and MSCs loading into the NICHE with collagen hydrogel as a carrier.

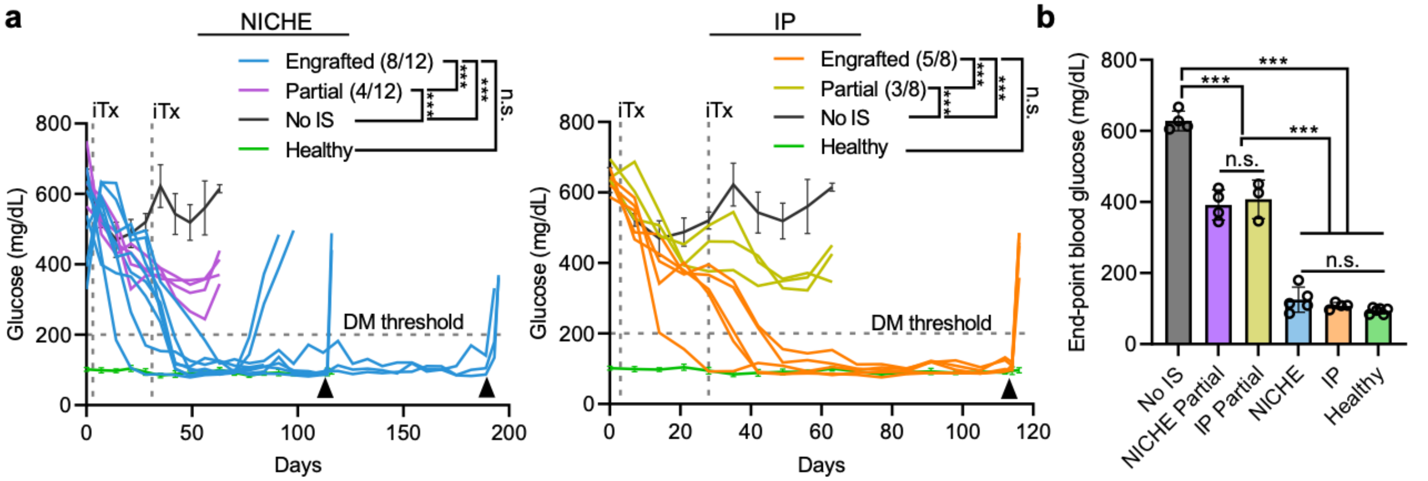

Supplementary Fig. 4 Blood glucose profiles after islet transplantation. a Blood glucose (BG) measurements of diabetic F344 rats transplanted with islets in NICHE cell reservoir. Individual BG plots for NICHE and IP rats with engrafted (diabetes reversal was achieved) and partial engraftment (glycemic benefit but no diabetes reversal achieved) are shown. No immunosuppression (No IS; *n* = 4 to day 63), and healthy controls (*n* = 6 to day 116, *n* = 1 to day 195) included for reference, mean ± SD. **b** BG at endpoint of No IS (*n* = 4 on day 63), IP partial (*n* = 3 on day 63), NICHE partial (*n* = 4 on

day 63), and healthy (*n* = 5 on day 115 and *n* = 1 on day 193); or prior to explant of NICHE engrafted (*n* = 2 on day 115, *n* = 3 on day 193) and IP engrafted (*n* = 4 on day 115). Mean ± SD, one-way ANOVA with Tukey's multiple comparisons test (n.s. $p = 0.9618$ NICHE versus IP; n.s. $p = 0.5768$ NICHE versus healthy; n.s. $p = 0.9792$ IP versus healthy; n.s. $p = 0.9842$ IP partial vs NICHE partial; ***$p<0.001$ No IS versus NICHE, IP, NICHE partial, IP partial, and healthy; ***$p<0.001$ NICHE partial and IP partial versus NICHE, IP, and healthy).

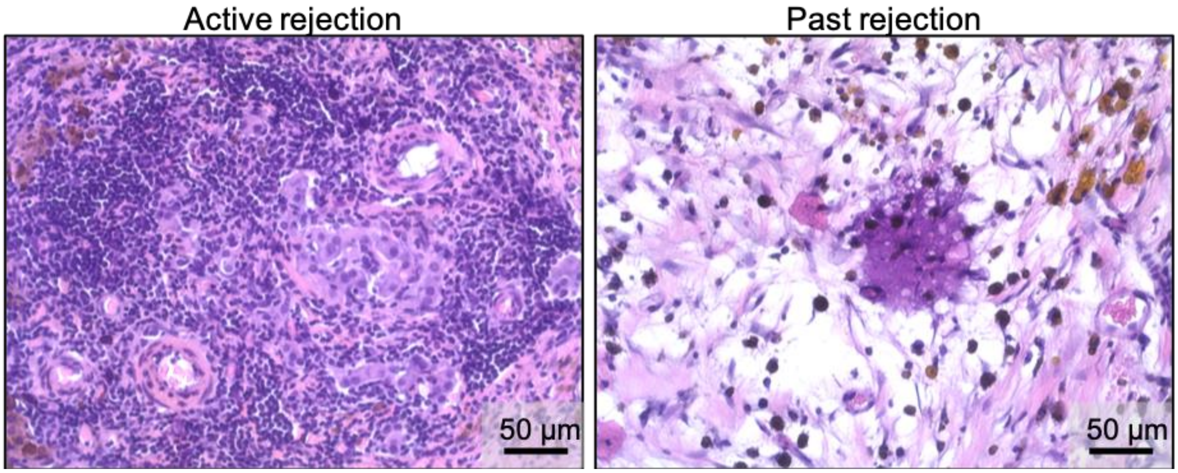

Supplementary Fig. 5 Rejected islet grafts in NICHE. Representative H&E micrographs showing islet grafts in NICHE with active (*n* = 3 biological replicates) and past rejection (*n* = 3 biological replicates).

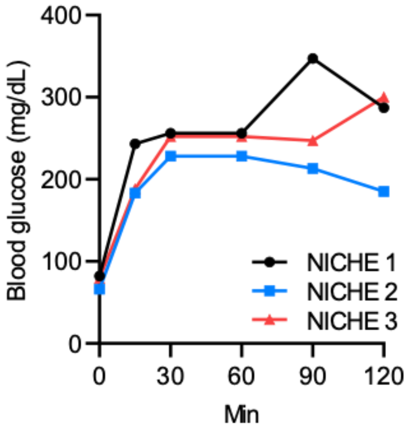

Supplementary Fig. 6 Graft destabilization. Blood glucose curves during intraperitoneal glucose tolerance test in NICHE rats on day 151 show graft destabilization.

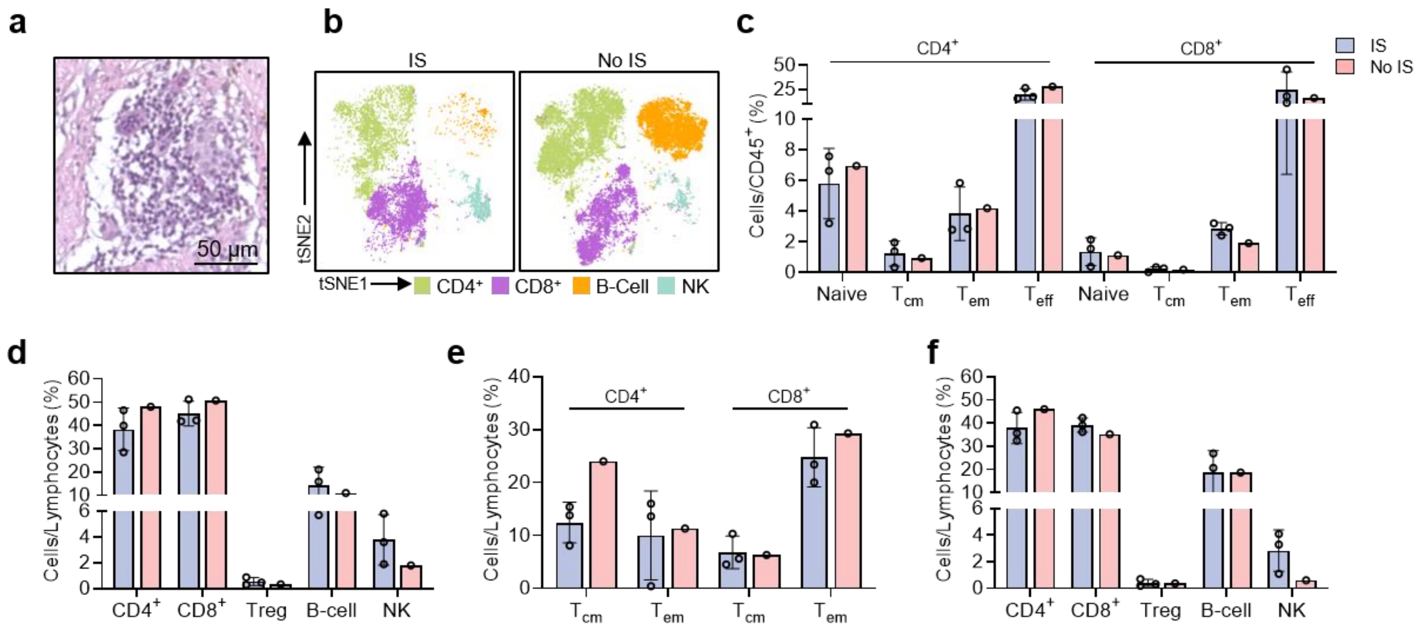

Supplementary Fig. 7 Mass cytometry (CyTOF) analysis of NICHE in nonhuman primates (NHPs). a Representative H&E staining of IS group cell reservoir tissue (*n* = 3 biological replicates) 14 days after islet transplantation showing leukocytic infiltration in the graft **b** tSNE plots of NHP CyTOF data. **c** CyTOF quantification of $CD4^+$ and $CD8^+$ memory T cell populations (*n* = 1 No IS and *n* = 3 IS independent NHP), mean ± SD. **d** Flow cytometry data of peripheral blood immune cell populations and **e** memory T cells 14 days after islet transplantation, and **f** of peripheral blood immune cell populations pre-transplantation (*n* = 1 No IS and *n* = 3 IS independent NHP), mean ± SD.

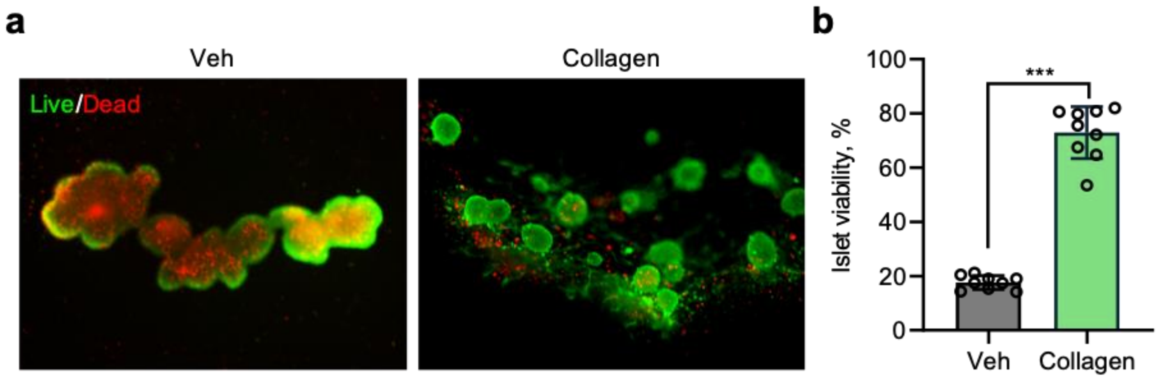

Supplementary Fig. 8 Islet viability in collagen matrix. a Live/Dead staining of islets after 5 days incubated in media (Veh) or collagen hydrogel used for transplantation and **b** quantification expressed as percent of live islet area (*n* = 9 biological replicates), mean ± SD, unpaired two-tailed student’s t-test ***$p < 0.001$.

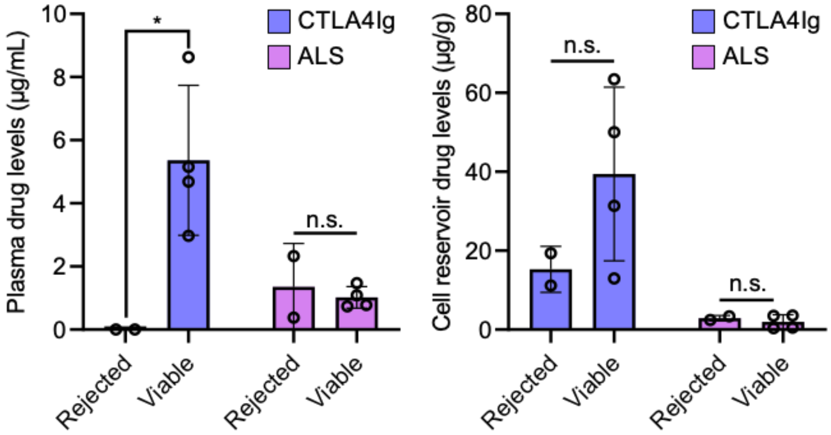

Supplementary Fig. 9 Immunosuppressant biodistribution in rats with viable and rejected grafts. Quantification of ALS and CTLA4Ig levels in plasma and cell reservoir tissue at endpoint of NICHE rats that rejected their grafts unexpectedly (n = 2) and rats that remained euglycemic with viable grafts (n = 4), mean ± SD, unpaired two-tailed student's t-test between viable and rejected grafts for each drug (*$p < 0.05$; n.s. $p = 0.6362$ Rejected vs viable plasma ALS; p = 0.2215 rejected versus viable cell reservoir CTLA4Ig; p = 0.5488 rejected versus viable cell reservoir ALS.

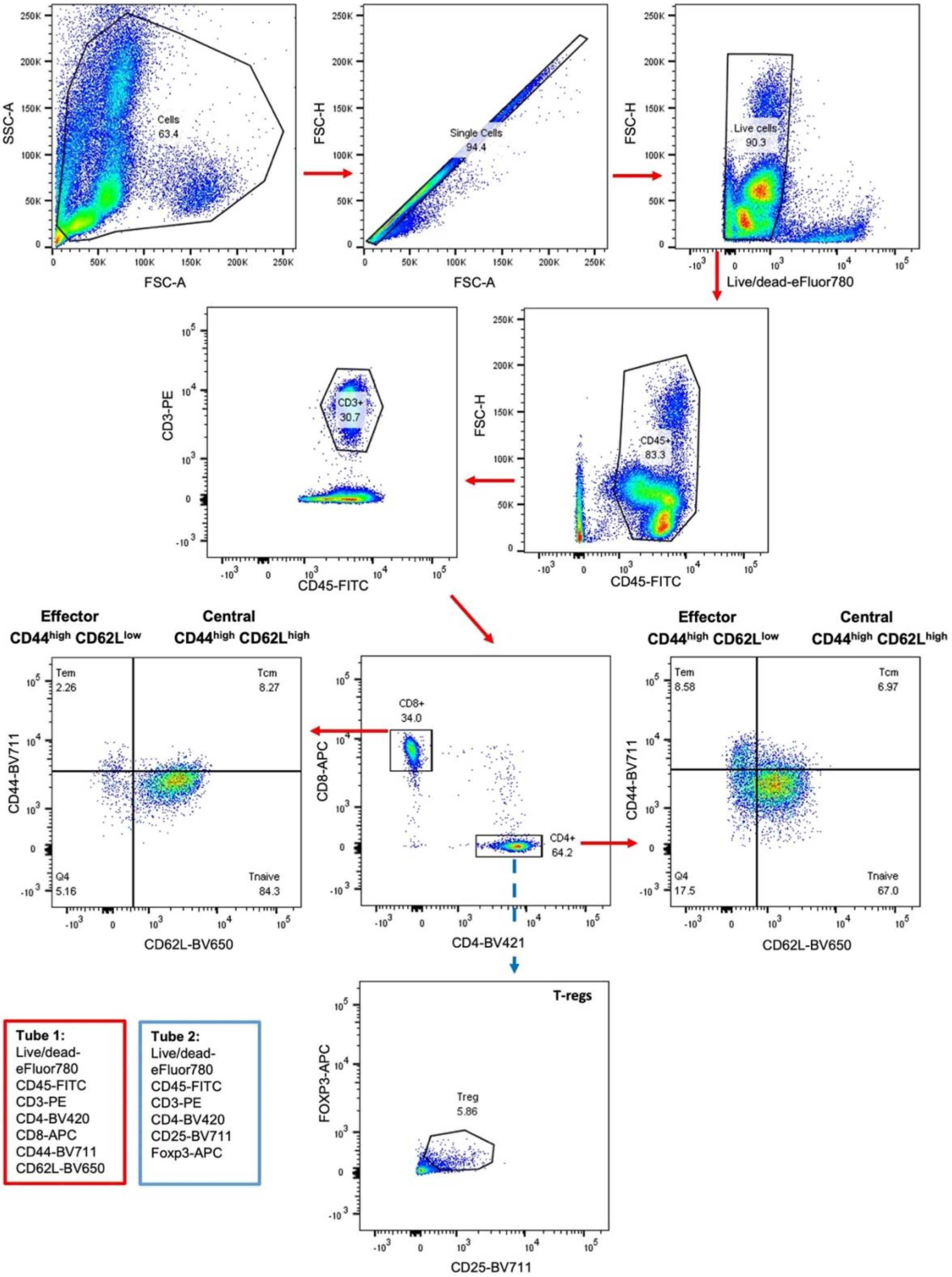

Supplementary Fig. 10 Gating strategy for flow cytometry data in rats. Red arrows show sequence. Cells were stained with two cocktails to assess memory T cells (Tube 1) and T-regs (Tube 2).

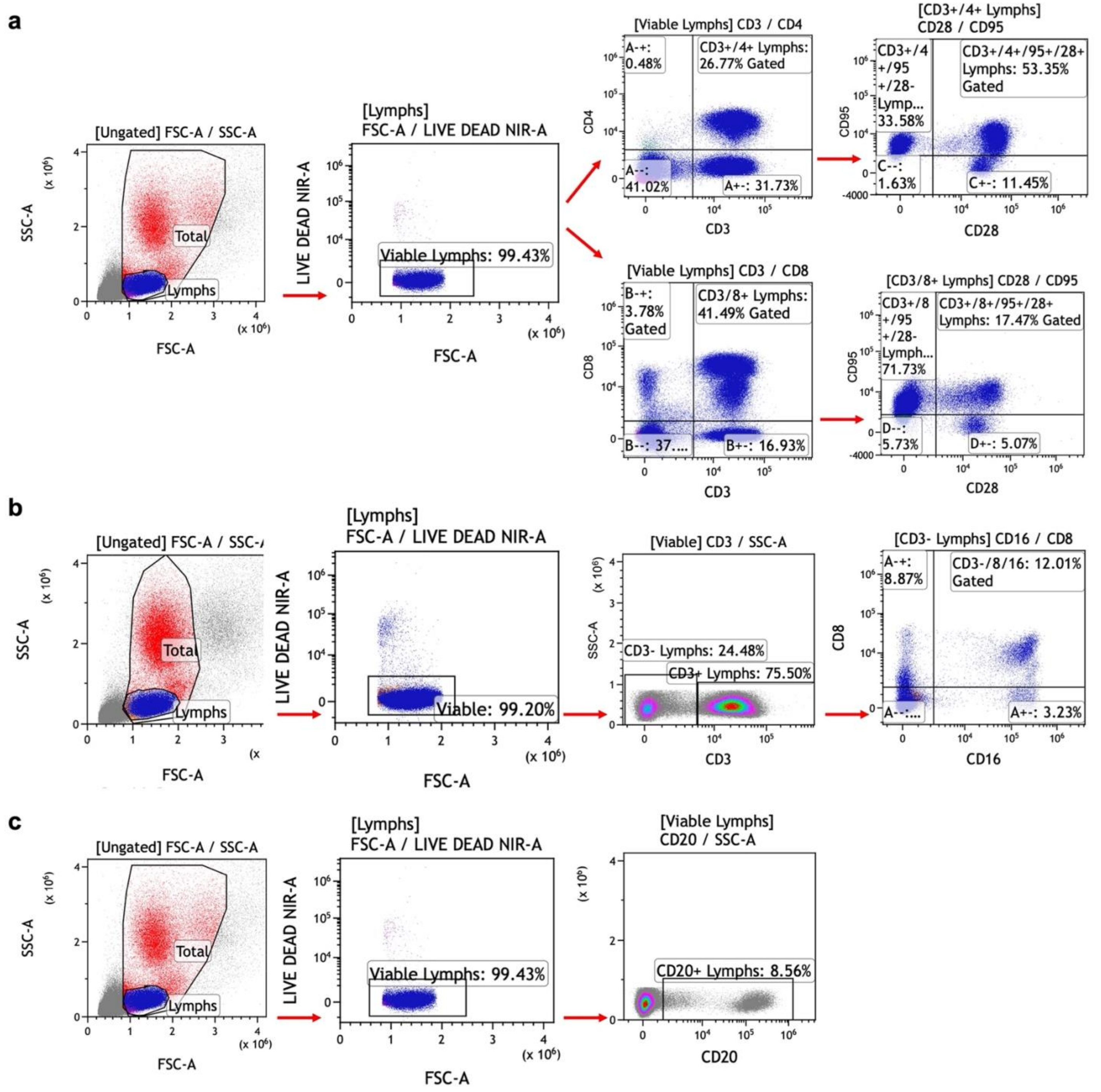

Supplementary Fig. 11 Gating strategy for flow cytometry data in NHP. Gating strategy for a) T-Cells, b) NK cells, c) B-Cells.

	Score					
**Cell characteristic**	0	1	2	3	4	Reactive inflammation multiplier ($m_{j,r}$)
Polymorphonuclear cells	0/HPF	Rare, 1-5/HPF	5-10/HPF	Heavy infiltrate	Packed	2
Lymphocytes	0/HPF	Rare, 1-5/HPF	5-10/HPF	Heavy infiltrate	Packed	2
Plasma cells	0/HPF	Rare, 1-5/HPF	5-10/HPF	Heavy infiltrate	Packed	2
Macrophages	0/HPF	Rare, 1-5/HPF	5-10/HPF	Heavy infiltrate	Packed	2
Giant cells	0/HPF	Rare, 1-5/HPF	5-10/HPF	Heavy infiltrate	Packed	2
**Tissue characteristic**						
Necrosis	0	Minimal	Mild	Moderate	Severe	2
Capsule thickness	0	Narrow (<5 cells)	Moderate (5-10 cells)	Thick (10-20 cells)	Extensive thick	1
Tissue inflammatory infiltrate	0	Minimal, focal in local tissue	Mild or multifocal in adjacent tissues	Moderate in adjacent tissues	Marked in adjacent tissues	1

Supplementary table 1. Scoring system used to assess implant reactivity. Reprinted with permission from Su et al.[1] under the Creative Commons Attribution 4.0 International license.

Gene	Assay ID
cdh5	Hs00901465_m1
nos3	Hs01574665_m1
vcam1	Hs01003372_m1
vegfa	Hs00900055_m1
gapdh	Hs02786624_g1

Supplementary table 2. List of TaqMan gene expression assay primers

Metal	Target	Make	Cat No.	Clone	Dilution
Gd156	CD45	Abcam	ab10558	-	1:100
Nd143	CD3	Cell Signaling	85061	D7A6E	1:100
Nd145	CD4	Abcam	ab237722	CAL4	1:100
Nd146	CD8	Invitrogen	MA1-70003	OX-8	1:100
Tb159	CD68	Biorad	MCA341GA	ED1	1:100
Yb171	Insulin	Cell Signaling	3014S	C27C9	1:100
Er168	Glucagon	Cell Signaling	2760	-	1:100
Sm147	Alpha smooth muscle actin	Invitrogen	MA5-11547	1A4	1:1000
Dy164	Myeloperoxidase	Dako	A0398	-	1:100
Er167	Ki-67	BD	550609	B56	1:100
Pr141	Granzyme B	Abcam	ab208586	EPR20129-217	1:100

Dy161	Arginase 1	Invitrogen	PA5-32267	-	1:100
Gd160	Foxp3	Invitrogen	700914	5H10L18	1:100

Supplementary table 3. Antibodies used for IMC

Metal	Target	Make	Cat No.	Clone	Dilution
Gd156	CD45	BD Bioscience	552566	D058-1283	1:200
Pr141	CD3	BD Bioscience	551916	SP34-2	1:200
Nd143	CD4	BD Bioscience	550625	L200	1:200
Nd146	CD8	Biolegend	301002	RPA-T8	1:800
Nd142	CD20	BD Bioscience	555621	2H7	1:200
Tm169	CCR7	Biolegend	353202	G043H7	1:200
Gd155	CD45RA	Biolegend	304102	HI100	1:400
Sm154	Foxp3	Biolegend	320201	259D	1:50
Yb176	CD56	Biolegend	318302	HCD56	1:100

Supplementary table 4. Markers used for CyTOF

Cell population	Gating strategy
CD4 T-Cell	$CD45^+CD3^+CD20^-CD4^+CD8^-$
CD8 T-Cell	$CD45^+CD3^+CD20^-CD4^-CD8^+$
Tem	$CCR7^-CD45RA^-$
Tcm	$CCR7^+CD45RA^-$
T-Naive	$CCR7^+CD45RA^+$
T-Effector	$CCR7^-CD45RA^+$
Treg	$CD45^+CD3^+CD20^-CD4^+CD8^-Foxp3^+$
B-Cell	$CD45^+CD20^+CD3^-$
NK	$CD45^+CD3^-CD20^-CD56^+$

Supplementary table 5. CyTOF gating strategy

Marker	Fluorophore	Clone	Make	Cat No.	Dilution
CD45	FITC	OX-1	BD Biosciences	561867	1:300
CD3	PE	G4.18	eBioscience	12-0030-82	1:300
CD4	BV421	OX-35	BD Biosciences	740040	1:300
CD8a	APC	OX8	eBioscience	17-0084-82	1:300
CD44	BV711	OX49	BD Biosciences	743924	1:300

CD62L	BV650	HLR1	BD Biosciences	743150	1:300
CD25	BV711	OX-39	eBioscience	742756	1:300
FOXP3	APC	FJK-16s	BD Biosciences	17-5773-82	1:50
Viability Dye	eFluor780	-	eBioscience	65-0865-14	1:100

Supplementary table 6. Antibodies used for flow cytometry in rat model.

Marker	Fluorophore	Clone	Make	Cat No.	Dilution
CD4	FITC	M-T477	BD Biosciences	556615	1 µg/test
CD28	PE	CD28.2	BD Biosciences	556622	1 µg/test
CD8	PerCP-Cy5.5	RPA-T8	BD Biosciences	560662	0.5 µg/test
CD3	PE-Cy7	SP34-2	BD Biosciences	557749	2 µL/test
CD16	APC	3G8	BD Biosciences	561248	1 µg/test
CD20	APC	2H7	BD Biosciences	559776	20 µL/test
CD25	BV421	BC96	Biolegend	302630	0.5 µg/test
FOXP3	APC	PCH101	eBioscience	17-4776-42	0.5 µg/test
CD95	PacBlue	DX2	Biolegend	305619	1 µg/test
Viability Dye	Fixable NIR	-	ThermoFisher	L10119	100 µL/test

Supplementary table 7. Antibodies used for flow cytometry in NHP model.

Supplementary methods

Assessment of cell permanence in NICHE

F344 BM-MSCs were harvested and resuspended at a density of 1 $x10^6$/mL. Next, cells were labeled with Vibrant DiD cell-labeling (Invitrogen) at a concentration of 5 µl/mL of cell suspension and incubated for 20 min at 37 ºC. Labeled cell suspensions were centrifuged and resuspended in 20% PF127 for further loading into NICHE cell reservoir following the same procedure to prepare devices described in the main methods section. Loaded NICHE (*n* = 3) were placed at 37 ºC to allow PF127-MSCs to gel. After 24 h incubation, NICHE fluorescent signal was imaged with IVIS. NICHE were then transferred to a neighboring empty well and both wells were imaged to assess cell leakage. Empty well and 20% PF127 were imaged as control.

Assessment of islet viability in collagen matrix

One-hundred Lewis rat islets were embedded in 100 µL of 4 mg/mL collagen matrix (RatCol, Advanced Biomatrix) and gelled in a 96-well plate at 37 °C. Once gelled, 100 µL supplemented culture media was added and incubated at 37 °C. Control islets were incubated in 200 µL supplemented culture media. After 7 days, samples were stained using LIVE/DEAD Viability/Cytotoxicity Kit (Invitrogen) and images captured using a BX61 Olympus microscope and analyzed with ImageJ software. Islet viability was calculated using Supplementary Eq. 1

$$\text{Islet viability } (\%) = \frac{\text{Live islet area}}{\text{Live islet area} + \text{Dead islet area}} * 100 \qquad (1)$$

Supplementary References

1 Su, J. T. *et al.* A Subcutaneous Implant of Tenofovir Alafenamide Fumarate Causes Local Inflammation and Tissue Necrosis in Rabbits and Macaques. *Antimicrob Agents Chemother* **64**, doi:10.1128/AAC.01893-19 (2020).
