# Supplementary material for: Implantable niche with local immunosuppression for islet allotransplantation achieves type 1 diabetes reversal in rats
Source: Nat Commun. 2022 Dec 26;13:7951. doi: 10.1038/s41467-022-35629-z (PMC9792517; doi:10.1038/s41467-022-35629-z)
Supplement: Supplementary file 3 — Reporting Summary [file 41467_2022_35629_MOESM3_ESM.pdf]

nature portfolio

Corresponding author(s): Alessandro Grattoni, PhD

Last updated by author(s): Nov 29, 2022

Reporting Summary

Nature Portfolio wishes to improve the reproducibility of the work that we publish. This form provides structure for consistency and transparency in reporting. For further information on Nature Portfolio policies, see our Editorial Policies and the Editorial Policy Checklist.

Statistics

For all statistical analyses, confirm that the following items are present in the figure legend, table legend, main text, or Methods section.

n/a	Confirmed	
☐	☒	The exact sample size (*n*) for each experimental group/condition, given as a discrete number and unit of measurement
☐	☒	A statement on whether measurements were taken from distinct samples or whether the same sample was measured repeatedly
☐	☒	The statistical test(s) used AND whether they are one- or two-sided<br>*Only common tests should be described solely by name; describe more complex techniques in the Methods section.*
☐	☒	A description of all covariates tested
☐	☒	A description of any assumptions or corrections, such as tests of normality and adjustment for multiple comparisons
☐	☒	A full description of the statistical parameters including central tendency (e.g. means) or other basic estimates (e.g. regression coefficient) AND variation (e.g. standard deviation) or associated estimates of uncertainty (e.g. confidence intervals)
☐	☒	For null hypothesis testing, the test statistic (e.g. *F*, *t*, *r*) with confidence intervals, effect sizes, degrees of freedom and *P* value noted<br>*Give P values as exact values whenever suitable.*
☒	☐	For Bayesian analysis, information on the choice of priors and Markov chain Monte Carlo settings
☒	☐	For hierarchical and complex designs, identification of the appropriate level for tests and full reporting of outcomes
☒	☐	Estimates of effect sizes (e.g. Cohen's *d*, Pearson's *r*), indicating how they were calculated

Our web collection on statistics for biologists contains articles on many of the points above.

Software and code

Policy information about availability of computer code

Data collection	NanoScope Analysis Software was used for roughness assessment. One Attension software was used for contact angle measurements. EVOS M5000 Imaging System Software, Keyence BZ-X800 Fluorescence Microscope System, and NIS-Elements Basic Research software were used for microscopy acquisition. StepOnePlus Real Time system was used for RT-qPCR data acquisition. Hyperion Imagin system was used for IMC data acquisition. Helios system software was used for CyTOF data acquisition. FACSDiva version 9 software was used for flow cytometry data acquisition. SolidWorks 3D CAD version 2021 was used to design the NICHE.
Data analysis	Tube length and number of tube segments were quantified using the Angiogenesis Analyzer plugin for ImageJ software. For IMC, data were segmented by ilastik and CellProfiler and histology topography cytometry analysis toolbox (HistoCAT) and R scripts were used to quantify cell number and generate tSNE plots as described in Schapiro, D et al. Nat Methods 14, 873–876 (2017). CyTOF data was analyzed with Cytobank using the gating strategy reported in methods. Flow cytometry data was analyzed with FlowJo version 10 software and Kaluza software version 1.5a. Graphpad Prism version 9 was used for data plotting and statistical analysis.

For manuscripts utilizing custom algorithms or software that are central to the research but not yet described in published literature, software must be made available to editors and reviewers. We strongly encourage code deposition in a community repository (e.g. GitHub). See the Nature Portfolio guidelines for submitting code & software for further information.

Data

Policy information about availability of data

All manuscripts must include a data availability statement. This statement should provide the following information, where applicable:

- Accession codes, unique identifiers, or web links for publicly available datasets
- A description of any restrictions on data availability
- For clinical datasets or third party data, please ensure that the statement adheres to our policy

The data generated in this study are provided in the Source Data file.

Human research participants

Policy information about studies involving human research participants and Sex and Gender in Research.

Reporting on sex and gender	Our study does not involve human research participants.
Population characteristics	See above
Recruitment	See above
Ethics oversight	See above

Note that full information on the approval of the study protocol must also be provided in the manuscript.

Field-specific reporting

Please select the one below that is the best fit for your research. If you are not sure, read the appropriate sections before making your selection.

☒ Life sciences ☐ Behavioural & social sciences ☐ Ecological, evolutionary & environmental sciences

For a reference copy of the document with all sections, see nature.com/documents/nr-reporting-summary-flat.pdf

Life sciences study design

All studies must disclose on these points even when the disclosure is negative.

Sample size	We used sample sizes comparable to other islet transplantation studies recently published in high-impact journals, including those at Nature Publishing Group (Wang, L.H., et al., 2021, Nat Commun 12, 5846; Song, W., et al., 2019, Nat Commun 10, 4602; Loh, K., et al., 2017, Nat Commun 8, 490. Tripathi, D., et al., 2016, Nat Commun 7, 13896.)
Data exclusions	No data were excluded.
Replication	All in vitro assays were performed at least twice to confirm reproducibility. Moreover, biological (where appropriate) and technical replicates were used during collection of all the data presented. This is specified for every technique/assessment in the methods section. Attempts at replication were successful.
Randomization	Cell culture-based assays used a common pool of cells or islets that were randomized (i.e. aliquoted) equally among treatment groups. For in vivo studies, rats were assigned a numerical identifier and randomized into experimental groups using the Research Randomizer software (randomizer.org) . Of note, animals were implanted with identical NICHE and rendered diabetic prior to randomization. For NHP studies, MHC haplotyping was performed via microsatellite analysis or deep sequencing on potential donor and recipient animals. Recipient animals were selected to ensure MHC I and II allele mismatch to the donor suitable to study transplant rejection. Once allocated in their groups, all recipient animals were handled identically.
Blinding	Scientists were blinded to group allocation during data collection and analysis. All samples were labeled with numerical identifiers (i.e. animal number rather than experimental group) and revealed until data interpretation.

Reporting for specific materials, systems and methods

We require information from authors about some types of materials, experimental systems and methods used in many studies. Here, indicate whether each material, system or method listed is relevant to your study. If you are not sure if a list item applies to your research, read the appropriate section before selecting a response.

Materials & experimental systems

n/a	Involved in the study	
☐	☒	Antibodies
☐	☒	Eukaryotic cell lines
☒	☐	Palaeontology and archaeology
☐	☒	Animals and other organisms
☒	☐	Clinical data
☒	☐	Dual use research of concern

Methods

n/a	Involved in the study	
☒	☐	ChIP-seq
☐	☒	Flow cytometry
☒	☐	MRI-based neuroimaging

Antibodies

Antibodies used

IHC in rats: Bandeiraea simplicifolia lectin (L2140, Sigma), CD31 (NB100-2284, Novus Biologicals), VE Cadherin (36-1900, Invitrogen), and eNOS (ab300071, Abcam), Anti-rabbit Alexa Flour 555 (A-21428, Invitrogen)
IHC NHP: CD31 (ab28364, Abcam), insulin (C27C9, Cell Signaling)
IMC: CD45 (ab10558, Abcam), CD3 (ab255972, Abcam), CD4 (ab237722, Abcam), CD8 (MA1-70003, Invitrogen), CD68 (MCA341GA, Biorad), Insulin (C27C9, Cell Signaling), Glucagon (2760, Cell Signaling), aSMA (, MPO (A0398, Dako), Ki.67 (ab231172, Abcam), GramZB (ab219803, Abcam), Arg1 (PA5-32267, Invitrogen), Foxp3 (ab238809, Abcam).
Flow cytometry rat: CD45 (OX-1, BD Biosciences), CD3 (G4.18, eBioscience), CD4 (OX-35, BD Biosciences), CD8a (OX8, eBioscience), CD44 (OX49, BD Bioscineces), CD62L (HLR1, BD Biosciences), CD25 (OX-39, eBioscience), FOXP3 (FJK-16s, BD Biosciences).
Flow cytometry NHP: CD4 (M-T477, BD Biosciences), CD28 (CD28.2, BD Biosciences), CD8 (RPA-T8, BD Biosciences), CD3 (SP34-2, BD Biosciences), CD16 (3G8, BD Biosciences), CD20 (BC96, Biolegend), FOP3 (PCH101, eBioscience), CD95 (DX2, Biolegend)

Validation

All antibodies were validated for the species and application used as stated in the product data sheet of each antibody provided by the manufacturer.

Eukaryotic cell lines

Policy information about cell lines and Sex and Gender in Research

Cell line source(s)

HUVEC were obtained from PromoCell (C-12200).
F344 rat MSCs were purchased from Cyagen (Cat No. RAFMX-01001). Lineage validation was reported by the manufacturer.

Authentication

For each cell line, cell lineage was authenticated via flow cytometric analyses for cell-type specific markers by the manufacturers and provided in the certificate of analysis.

Mycoplasma contamination

All cell lines tested negative for mycoplasma

Commonly misidentified lines
(See ICLAC register)

The cell lines purchased for this study were checked against and not found in the list of known misidentified cell lines maintained by the International Cell Line Authentication Committee.

Animals and other research organisms

Policy information about studies involving animals; ARRIVE guidelines recommended for reporting animal research, and Sex and Gender in Research

Laboratory animals

8-week-old Fischer 344 rat; 8-week-old Lewis rat; Mauritian cynomolgus macaque (Macaca fascicularis) age 5 years old; Vietnamese cynomolgus macaques (n = 6) ages 11, 11, 12, 12, 13, and 14 years old.

Wild animals

The study did not involve wild animals.

Reporting on sex

Male rats were used as they develop more robust STZ-induced hyperglycemia. For NHP studies, a male mauritian cynomolgus macaque was used as the donor and female vietnamese cynomolgus macaques served as recipients. For these studies, MHC mismatching was the deciding factor for macaque selection from a pre-existing pool of available NHP.

Field-collected samples

The study did not involve samples collected in the field.

Ethics oversight

For rat studies, all procedures were approved by the Houston Methodist Institutional Animal Care and Use Committee (IACUC) in accordance with the National Institute of Health Guide for the Care and Use of Laboratory Animals and the Animal Welfare Act.
For NHP studies, all experiments were carried out according to the provisions of the Animal Welfare Act, PHS Animal Welfare Policy, and the principles of the NIH Guide for the Care and Use of Laboratory Animals. All procedures were approved by the IACUC at The University of Texas MD Anderson Cancer Center and University of Miami.

Note that full information on the approval of the study protocol must also be provided in the manuscript.

Flow Cytometry

Plots

Confirm that:

☒ The axis labels state the marker and fluorochrome used (e.g. CD4-FITC).

☒ The axis scales are clearly visible. Include numbers along axes only for bottom left plot of group (a 'group' is an analysis of identical markers).

☒ All plots are contour plots with outliers or pseudocolor plots.

☒ A numerical value for number of cells or percentage (with statistics) is provided.

Methodology

Sample preparation	For memory T cell analysis at endpoint, spleens were disaggregated into single cell suspensions via mechanical dissociation in PBS with 5 mM EDTA and filtered through a 40 µm cell strainer. Red blood cells were removed via incubation with ACK lysis buffer (Quality Biological). Cells were washed and, following incubation with FC blocker (αCD32, BD Biosciences), 1 x 106 cells were stained with CD45, CD3, CD4, CD8a, CD44, CD62L, and viability dye. Cells were washed with 2% FBS in PBS with 5 mM EDTA and fixed with IC fixation buffer (Invitrogen). For blood analysis, 100 µL of whole blood anticoagulated with EDTA was stained with same markers as the spleen followed by incubation with 1-step Fix/Lyse Solution (eBioscience). Cells were transferred to 5 mL FACS tubes with 40 µm cell strainer (Corning) and washed twice with 2% FBS in PBS. For circulating lymphocyte analysis, 100 µL whole blood was stained as above with CD45 and CD3. For circulating Treg analysis, 100 µL of whole blood was stained as above with CD45, CD3, CD4, CD25, and viability dye, followed by fixation with eBioscience fixation/permeabilization buffer (Invitrogen), permeabilization with 1X permeabilization buffer (Invitrogen), and intracellular staining with Foxp3. Cells were washed twice and re-suspended in 2% FBS in PBS with 5 mM EDTA.
Instrument	Data was acquired on LSRII flow cytometer (BD Biosciences).
Software	Data was acquired with FACSDiva software and analyzed with FlowJo v10 software or Kaluza software v1.5a.
Cell population abundance	At least 100,000 events were recorded for each sample in the "cells" gate. We obtained >90% singlets and 80-90% cell viability.
Gating strategy	For Rats: General population was first plotted in FSC, SSC gate and cells discriminated from debris based event size and distribution. Next, cells were gated on FSC-A vs FSC-H plots to select single cells. Then, live cells were gated from the viability dye channel (negative for stain). Leukocytes were determined as CD45 positive cells in a CD45 vs FSC plot. Lymphocytes were then subgated from the CD45+ population in a CD3 vs FSC plot. Next, CD4 and CD8 lymphocytes were gated in a CD4+ and CD8+ plot. For each one, CD44 and CD62L plot was used to gate Tcm (CD44hiCD62Lhi) and Tem (CD44hiCD62lo) populations. T regs were gated from a CD25 vs Foxp3 plot taken from the CD4+ population.<br><br>For NHP: Mononuclear cells were gated from general population in a FSC-A vs SSC-A plot. Next, live cells were gated from the viability dye channel (negative for stain). CD4 and CD8 lymphocytes were gated in a CD4 vs CD3 and CD8 vs CD3 plot. For each one, Tcm (CD95+CD28+) and Tem (CD95+CD28-) were subgated from a CD95 vs CD28 plot. For NK cells, following gating of live mononuclears, CD3- cells were selected from a CD3 vs SSC-A plot. Next, CD16+CD8+ cells were subgated from a CD16 vs CD8 plot. B cells were gated as CD20+ cells from live mononuclear plot. T regs were gated from a CD25 vs Foxp3 plot taken from the CD4+ population.

☒ Tick this box to confirm that a figure exemplifying the gating strategy is provided in the Supplementary Information.
